# Modern causal inference approaches to investigate biodiversity-ecosystem functioning relationships

**DOI:** 10.1038/s41467-023-37546-1

**Published:** 2023-04-06

**Authors:** Jakob Runge

**Affiliations:** 1 Deutsches Zentrum für Luft- und Raumfahrt (DLR), Institut für Datenwissenschaften, Mälzerstr. 3-5, Jena, 07745 Germany; 2 Technische Universität Berlin, Institute of Computer Engineering and Microelectronics, Straße des 17. Juni 135, Berlin, 10623 Germany

**Keywords:** Ecology, Environmental sciences, Biodiversity

## Abstract

Detecting and quantifying the causal relations of ecosystem functioning is a challenging endeavor. This Comment discusses a global study on grasslands and illustrates how reasoning about underlying assumptions is key to modern causal inference approaches in ecology.

## Teaser

Detecting and quantifying the causal relations of ecosystem functioning is a challenging endeavor. A global study on grasslands illustrates how reasoning about underlying assumptions, from confounding and nonlinearity to fundamental questions of determinism, is key to unlocking the potential of modern causal inference approaches in ecology.

How does biodiversity effect ecosystem productivity? How much diversity does an ecosystem need? Beyond their academic interest, these questions are at the heart of understanding the consequences of largely human-induced changes in biodiversity.

At its core, this is a causal question –predicting the effect of an intervention in species richness on ecosystem functioning–, as opposed to passively observing both: Some measure of correlation would indeed partly measure the causal effect of richness on productivity, but both are also caused by many other factors, for instance, precipitation, and such confounders would also contribute to their correlation.

There are two approaches to answer causal questions: The first one is to actually intervene via the experimental design, where one would randomly select plots to manipulate them with different levels of species richness and then measure how productivity changes at the treated versus the untreated plots. This reduces confounding by generating plots that are comparable with respect to known and unknown confounding factors. However, experiments are generally costly, potentially unethical, and in this case a crucial question is how to intervene: If the causal effect of richness on productivity depends on the specific changes of species, experimental inferences may not generalize to natural ecosystems^1^.

These challenges have led to the development of a second approach in recent decades: To utilize domain assumptions about the underlying system to infer causal effects from observational data within the rich frameworks of causal inference. Two main frameworks are the graphical-causal-model framework^2,3^ and the potential-outcome framework^4,5^. Both frameworks are equivalent from a theoretical point of view, but they differ in how assumptions are stated: The potential outcome framework uses an algebra of counterfactuals, while the graphical-causal-model framework uses, well, graphical causal models. These are directed acyclic graphs where the nodes indicate the relevant processes of the system and the arrows indicate direct causal relations. For an introduction to causal inference on time series data see^6^.

**Fig. 1:**
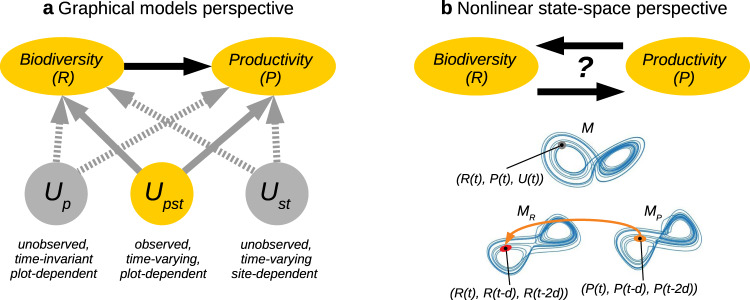
Two perspectives on causal inference in ecology. **a** Graphical models perspective, where a researcher would assume qualitative causal relationships in the form of a directed acyclic graph among the variables of interest, in Dee et al.^11^ biodiversity richness *R* and ecosystem productivity *P*, as well as confounding variables *U*. In the specific fixed-effects panel regression setting of Dee et al.^11^ the causal effect of *R* on *P* can be estimated with a statistical model such as eq. (1) that includes dummy variables to account for unobserved confounders. These can either be time-invariant and only vary across plots (*U*_*p*_), or time- and site-varying, but approximately the same for different plots (*U*_*st*_). However, confounders that vary across plots, sites, and time (*U*_*pst*_) can only be accounted for if they are observed and explicitly included in the model (not used in eq. (1)). **b** Nonlinear state-space perspective where the primary goal is to detect whether and in which direction causation exists. A method like convergent cross-mapping (CCM) assumes an underlying deterministic nonlinear system with attractor *M* that can be reconstructed from the variables' state spaces (*M*_*R*_, *M*_*P*_) using time-lagged coordinate embedding. CCM concludes on *R* → *P* if points on *M*_*R*_ can be predicted using nearest neighbors in *M*_*P*_ (orange ellipse) and the prediction improves the more points on the attractor are sampled. Implicitly, CCM makes the assumption that a successfully reconstructed state-space includes the influence of observed or unobserved confounders *U*.

A typical observational causal study would start with building a statistical model of species richness and ecosystem productivity, as the naive correlation approach mentioned above, but then add qualitative domain knowledge, for example, in the form of a causal graph, to read off which confounding variables to include in the model such that these block confounding mechanisms (Fig. 1a). However, to fully eliminate confounding effects, observational causal study designs in ecology face the daunting task of identifying, measuring, and statistically controlling for a myriad of confounding variables, from local weather to land-use history^7^.

This can be overcome if multiple time-series (or longitudinal) datasets of the system, for example, at different sites, are available. Then one can jointly leverage these to circumvent observed, as well as unobserved confounding. Multiple datasets are often available in social sciences, where they are called panel data, and the trick then is to include dummy parameters in the statistical regression model, termed fixed-effects panel regression.

Fixed-effects panel regression was mostly developed within the potential outcome framework^4,5^, but the graphical models framework is catching up^8,9^. On the other hand, explicating assumptions in the form a causal graph is arguably more intuitive than an algebra of counterfactuals, but we will not enter the partially tense discussions between the two factions here^10^.

## Integrating causal graphs in fixed-effects panel regression for a global grassland study

Enter the new study by Dee et al.^11^, which aptly utilized both causal graphs and panel regression to systematically study the effect of richness on productivity from longitudinal data (2007-2017) of 151 unmanipulated plots in 43 grassland sites across 11 countries (Fig. 1a). The authors’ goal was to quantify the causal effect of plant richness *R* on biomass productivity *P* given panel time series data (indexed with *t*) at different sites *s* and plots *p*. The fixed-effects regression model then is

1 $$\ln ({P}_{pst})=\beta \cdot \ln ({R}_{pst})+{\delta }_{p}+{\mu }_{st}+{\epsilon }_{pst}.$$

The ln-ln specification is used because the effect of richness on productivity is assumed to be non-linear, but more on nonlinearity later. The regression coefficient *β* now quantifies the causal effect of interest, the expected percent change in productivity given a one percent change in richness. *β* is assumed to be independent of time *t*, sites *s*, and plots *p*, and the fixed-effects trick is to add the plot-dummy parameter *δ*_*p*_ and the site and time-dummy parameter *μ*_*st*_ to model any time- or plot or site-specific effect and apply the model on the pooled data (all time series concatenated) to estimate *β*, *δ*_*p*_, and *μ*_*st*_. The plot-dummy *δ*_*p*_ has a similar effect as de-meaning the plot time series and, hence, removing any time-invariant confounding effect specific to each plot, while the site and time-dummy *μ*_*st*_ removes confounding in the site and time-dimension. Any remaining variance is then captured in the noise term *ϵ*_*pst*_.

Contrary to expectations (see^12,13^), the authors find that higher plot-level richness causes productivity to decline (that is, *β* < 0) rather than increase. The authors attribute the differences to observational studies to prior work not controlling for enough confounding factors, and the difference to experimental studies to the challenge that these had planted fewer rare and non-native species than exist in nature (the challenge to design “natural" interventions). While increases in native species enhance productivity, increases in rare species reduce productivity, which explains the difference.

Panel regression is not a silver bullet. Besides more technical statistical assumptions^5^, the method only accounts for two types of confounders: Those that are essentially invariant over the study period, in Dee et al.^11^, for example, topography or soil type, and those that vary by both site and year, but are largely invariant over the different plots at a site, such as the surrounding land-use change. This leaves open potential confounding effects due to processes that are plot-level specific and time-varying.

To address this issue, Dee et al.^11^ went one step further and investigated other model designs to see whether their conclusions are robust, from an instrumental variable design and mediation analysis to accounting for time-lagged dependencies. This is an important step for transparent causal inference^6^: Rather than avoiding explicit causal language, it encourages the researcher to explicitly lay out assumptions that enable more robust conclusions and to discuss conclusions under alternative sets of assumptions. This explicit interpretability is also one of the reasons why causal inference is now becoming a pillar of modern AI^10^.

## Alternative assumptions towards inferring causation in ecology

The above discussion was focused around overcoming confounding in estimating a specific causal effect by utilizing qualitative assumptions about how the cause and effect in the system are related to potential confounding processes. In the graphical causal models framework these assumptions are explicated in a causal graph whose nodes are random variables of an underlying structural causal model. Each variable’s next observation is determined by an assignment function (or autonomous causal mechanism) of the node’s parents and an exogeneous noise term that subsumes the effects of all factors that are not part of the model and unique to that node. Hence, these noise terms are mutually independent, an assumption called the Causal Markov Condition^2^.

This underlying systemic view then leads to the “intervention-based” notion of causality, which assumes that, in principle, the causal variable can be manipulated, either by humans or nature, by “replacing" its causal mechanism by the intervention value (for example, a certain plant species richness) leading to the causal *do*-calculus^2^.

But there is yet another framework of causality: the prediction-oriented convergent-cross mapping (CCM) approach assumes an underlying deterministic dynamical system^14^ (Fig. 1b). CCM is mainly used for what is called causal discovery, also a hallmark of the causal graphical model framework^15^, that is, to detect causation, rather than assume it qualitatively exists and quantify it, as discussed above. However,^16^ derived the empirical dynamic modeling (EDM) framework from CCM.

Both CCM and the graphical models (or potential outcome) framework have their distinct sets of assumptions and current capacities of dealing with various challenges. In the original paper^14^, CCM was contrasted against linear Granger causality^17^. CCM utilizes nonparametric methods that make few assumptions about the underlying functional dependencies and can well model highly nonlinear dynamics. However, CCM also crucially assumes the existence of an (ideally low-dimensional) attractor, exemplified on the Lorenz attractor in ref. ^14^, and the delicate task is then to reconstruct it using delay embedding^18^. CCM makes the somewhat optimistic assumption that, by successfully reconstructing the attractor, the whole state space, including the influence of observed or unobserved confounders, is implicitly reconstructed. If such a low-dimensional (and ideally not very noisy) attractor is not reconstructable, then the CCM framework cannot well distinguish confounding from causation, as several studies have shown^19,20^.

These findings illustrate that the theory of CCM on dealing with confounding of the various forms treated in this article requires further development. But there have been advances in integrating ideas from causal inference into CCM. Extensions of CCM include dealing with time-delayed causal interactions^21^, making it more robust^22^, (partially) accounting for confounders^23^, but also handling multiple datasets as in panel regression^24^. However, the spatial CCM variant for the latter task cannot well handle heterogenous dynamics among sites and relatively short time series.

While the causal graphical model and potential outcome frameworks are, in principle, non-parametric and can be combined with machine learning for nonlinear causal effect estimation^25^, the field, starting out from social sciences, has yet to fully embrace the complexity of nonlinear dynamical systems and time series^6^.

In summary, for method developers there is ample room and opportunity to join ideas from different perspectives. More workshops and joint papers from scientists of the different communities could pave the way to such integration. For those wishing to apply causal inference methods to ecology, Dee et al.^11^ impressively demonstrate on complex ecosystem interactions how to make assumptions transparent and integrate causal reasoning into data-driven science.
